# Research on Niche Improvement Path of Photovoltaic Agriculture in China

**DOI:** 10.3390/ijerph192013087

**Published:** 2022-10-12

**Authors:** Lingjun Wang, Yuanyuan Li

**Affiliations:** 1 School of Economics and Management, Nanjing Institute of Technology, Nanjing 211167, China; 2 NJIT Institute of Industrial Economy and Innovation Management, Nanjing 211167, China; 3 School of Food Science, Nanjing Xiaozhuang University, Nanjing 211171, China

**Keywords:** photovoltaic agriculture, niche improvement path, niche influencing factors, DANP, NK model

## Abstract

To explore the niche improvement path of photovoltaic agriculture in China, a niche influencing factor system was constructed first. Then, this study innovatively combined the DEMATEL and analytic network process (DANP) method and the NK model, which can correct the defects of the traditional NK model. Based on the above method, the influence coefficients and index weight of each niche factor were calculated, and the niche fitness landscape of photovoltaic agriculture was constructed. Finally, according to the fitness landscape map of each combination state, the optimal configuration state of niche influencing factors of photovoltaic agriculture and the optimal niche improvement path of photovoltaic agriculture were explored. We found that the interaction between the six niche influencing factors determines the niche fitness of photovoltaic agriculture, and the changes in the niche fitness and the niche improvement of photovoltaic agriculture are coordinated. It was proposed that the optimal niche improvement path of photovoltaic agriculture in China is “technological innovation → policy formulation → resource allocation → economic improvement → social recognition → environmental protection”, and the research conclusions were further explained and discussed.

## 1. Introduction

It has become a broad global consensus to vigorously develop renewable energy sources, effectively respond to climate change, and promote the transition to clean and low-carbon energy sources [1]. Due to the characteristics of safety and convenience, the utilization of solar energy resources has attracted the attention of various countries [2,3]. Among them, photovoltaic power generation is the most common form. Thanks to policy support, China has established a complete photovoltaic industry chain [4] during the “12th Five-Year Plan” period, and has become a leader in the production and application of photovoltaic products [5]. Moreover, innovative modes of coupled development of photovoltaic power generation and agricultural planting, photovoltaic power generation and agricultural breeding, and photovoltaic power generation and ecological governance emerged during this period. During the “13th Five-Year Plan” period, China National Energy Administration issued the *13th Five-Year Plan for Solar Energy Development*, and the Ministry of Industry and Information Technology and other departments of China jointly issued the *Intelligent Photovoltaic Industry Development Action Plan (2018–2020)*. These two plans proposed the integrated development of photovoltaics and agriculture, expanded the integration of photovoltaic applications in agriculture, and promoted the development of photovoltaic agriculture in China to some extent. In 2021, the State Council of China proposed to take the lead in promoting clean energy and ensure a good job in the integrated development with agriculture in the *Guidance on Accelerating the Establishment and Improvement of the Economic System for Green, Low-Carbon and Circular Development*. It can be predicted that photovoltaics, as an important part of clean energy, has a huge development space for its integration with agriculture, and photovoltaic agriculture has broad prospects for development.

In 2011, photovoltaic agriculture began as a global project practice [6], and now the number of agriculture photovoltaic projects in the world exceeds 2200 [7]. At the same time, since 2017, the governments of Japan, France, Massachusetts, South Korea, and China have introduced relevant policies in the application and promotion of photovoltaic agriculture. The photovoltaic agriculture market in these countries has already taken the lead in other regions, among which the scale of China is to the maximum. According to the statistics of this study, by the end of 2020, the installed capacity of grid-connected photovoltaic agriculture projects in China accounts for about 7% of the total installed photovoltaic power capacity. According to the *2035 National Photovoltaic Development Research Report* predicted by the Energy Internet Innovation Research Institute of Tsinghua University, China’s total installed photovoltaic capacity will reach 1500 GW in 2035, about six times that of 2020. It can be predicted that in the future, more photovoltaic power stations will be built on agricultural land, and the integrated development of photovoltaic and agriculture will also become an inevitable trend. How to better integrate photovoltaics and agriculture, so as to establish an efficient clean energy–food system, and then promote the sustainable development of photovoltaic agriculture, is of important and far-reaching significance for the realization of China’s targets of carbon peak and carbon neutrality based on ensuring food security.

Photovoltaic agriculture is unique to China. Chinese scholars generally define it as a special form of agriculture, such as a comprehensive agricultural production system which can achieve economic and social development, ecological environment protection and efficient utilization [8], an important form of agricultural engineering [9], or a high-technology agricultural industry [10]. When studying the combination of agriculture and photovoltaics, scholars from other countries generally divide it into the application of photovoltaics in agriculture and agrivoltaic. The latter refers to the photovoltaic power generation and agricultural production on the same land, which emphasizes the “dual use in one place”. It can be seen from the above that photovoltaic agriculture proposed by Chinese scholars not only contains the idea of “one land for dual use”, but also includes the application of photovoltaics in agriculture.

The application of photovoltaics in agriculture first appeared in agricultural irrigation, and the first photovoltaic water pump was launched in 1975. In the early 1980s, the Lewis Research Center of NASA launched a global market forecasting study on the use of photovoltaic systems in the agricultural sector in Plan 1980–1981, selecting Nigeria, Morocco, Colombia, Mexico, and the Philippines for the economic importance, energy status, electrification, solar resources, and geographical representation. It then analyzed the obstacles of international marketing of American photovoltaic products and proposed relevant suggestions [11]. In the following period, scholars mainly focused on the barriers to the application of solar photovoltaics in agriculture, which mainly came from the economic, institutional, and social aspects [12]. Because of these barriers, there was very little literature on the application of photovoltaics in agriculture in the 1990s. Some scholars believe that the best time for photovoltaics application in agriculture should be after 2000 [13]. The problem of barriers to the application of PV in agriculture was still mentioned in the early-stage literature in the 21st century, including Radulovic’s proposal to promote the application of PV in the Indian agricultural sector, proposing to overcome “political barriers” [14]. Mousazadeh also analyzed the issue of “replacement cost barriers” in related research [15]. However, many scholars have optimistic expectations about the application of photovoltaics in agriculture, believing that the development and utilization of solar energy can promote global sustainable agriculture and rural development, and have good application prospects in intelligent ecological agriculture [16]. At the same time, in addition to the application in agricultural irrigation, the application of photovoltaics in agriculture shows a diversified trend, including agricultural greenhouse systems, agricultural products storage, and agricultural electric vehicles and farm lighting [17,18]. Table 1 shows the development history of photovoltaics application in agriculture.

Different from the application of photovoltaics in agriculture, agrivoltaics pays more attention to the mutual influence, the competition and cooperation relationship, and the coupling and symbiosis of photovoltaic power generation and agricultural production. The idea that solar conversion and crop cultivation could coexist (coexistence) was first proposed in 1982 [19]. It took about 30 years for this idea to be expressed as “agrivoltaics”; since then, the large-scale practice has started around the world [6]. Throughout the research in this field, scholars mainly carried out research from two aspects of agriculture and photovoltaic engineering technology. In agriculture, the representative scholars are Dupraz and Marrou of the French National Academy of Agricultural Sciences (INRA), by whom the above concept of “agrivoltaics” was first proposed. They conducted a comprehensive study on the growth of crops in agrivoltaic systems [20,21], providing a theoretical basis for the application and follow-up research of agrivoltaic systems. They found that the coupling between agricultural farming and photovoltaic power can be efficient, but it needs to be optimized, namely, a balance between agricultural production and photovoltaic power generation, to form a better collaboration between the two. In terms of photovoltaic engineering technology, the representative scholars are Goetzberger, Trommsdorff, and Schindele of the Fraunhofer Institute for Solar Energy Systems in Germany. Among them, Goetzberger pointed out that these photovoltaic systems occupy only one-third of the land and light resources, and further technical improvements can improve their applicability in crop production [19]. Trommsdorff conducted the first comprehensive technical and economic analysis of agrivoltaics, extending the research of agrivoltaics to the economic field. He regarded agrivoltaics as hybrid technology, constructed a research model based on the neoclassical theory of welfare economics, and derived the technical efficiency standard of agrivoltaics. Schindele promoted the research of agrivoltaics to the direction of social economy. He carried out the technical economic analysis of agrivoltaics with the indicators designed, and proposed the relevant policy enlightenment on this basis [7]. In addition, similar to the application of photovoltaics in agriculture, scholars believe that agrivoltaics has good application prospects. This dual land use method can both meet the social demand for clean energy and protect the agricultural production land, which many countries and regions are happy to accept [22,23,24]. At the same time, the specific implementation of agrivoltaic projects will involve issues about social recognition, which is also a concern of some recent research institutes [25,26].

It can be seen from the above that the research of photovoltaic agriculture has expanded from the early technology application field to the economic, social, and other fields. In addition, since the technology of photovoltaic agriculture already involves the utilization of land and light resources, and since photovoltaic power generation can be also locally applied to agricultural production, its environmental and ecological impacts have always been more investigated by scholars [27,28]. However, most of the current research stays at the micro level of case analysis [29,30,31], and there is a lack of macro research on the overall situation of photovoltaic agriculture in a country or a certain region. Due to the lack of official statistical information on the development of photovoltaic agriculture, the government lacks understanding of the specific implementation of photovoltaic agriculture. It is impossible to formulate an appropriate management plan, improve its agricultural sustainability on a large scale, and assess the land consumption and the environmental, economic, and social impact on the region [32].

Niche belongs to the category of ecology and is an important concept of modern ecology. Ecologists use niche to express the spatial range of biological habitats, and they explain the existence and competition of organisms in the environment with this theory. In general, niche mainly reflects the resource occupation and functional position of a biological unit in the ecosystem [33]. Similar to ecology, niche theory is not only widely used in natural science, but is also introduced into social science research, especially in the fields of economy [34,35,36] and management [37,38,39]. As the basic unit of the ecosystem, the ecological niche is the platform for the communication between the organization and the environment, and the competition among the organizations revolves around the competition for the ecological niche. Organizations establish relationships with other organizations by virtue of their own niches, and show certain differences based on the niches they occupy. Niche theory pays attention to the choice and influence of the environment on the organization. It has a great reference for the survival and development of the organization, and can provide a correct angle and direction for the study of organizational problems.

The ecological niche of photovoltaic agriculture refers to the space scope and resources occupied by the organization at a certain time and in the environment, which essentially reflects the state and function of photovoltaic agriculture in a specific ecological environment. The study of the ecological niche of photovoltaic agriculture is conducive to clarifying the interactive relationship between photovoltaic agriculture and its environment in the development process, and to clarifying the development trend and development stage of photovoltaic agriculture. It helps to define development goals and provide policy guidance to regulators. According to Wang, the ecological niche of China’s photovoltaic agriculture will undergo a four-stage evolution process: positioning, integration, leap, and symbiosis [40]. China has completed the positioning stage and entered the integration stage. Based on the above research, this paper explores the specific improvement path of the ecological niche of China’s photovoltaic agriculture, which is a further expansion of the above research, and also provides theoretical support for the sustainable development of China’s photovoltaic agriculture.

To explore the niche improvement path of photovoltaic agriculture, the niche influencing factor system of photovoltaic agriculture was constructed. The impact of these factors on the niche level of photovoltaic agriculture is different, so the impact of each factor, that is, the weight, needs to be determined. There are many ways to determine weights; the DANP method is a weighting method that combines the analytical network process (ANP) and DEMATEL methods. In 1996, Professor T. L. Saaty of the United States proposed the ANP method [41]. This method evolved from the analytic hierarchy process (AHP). The advantage of the ANP method over AHP is that this method considers the network relationship of mutual influence between indicators, and avoids the drawbacks of independent indicators in the AHP method. The DEMATEL method is widely used in analyzing the relationship between indicators and solving the problem of interdependence between elements [42]. By analyzing the influence and degree of influence between factors, this method can calculate the centrality and causality of each factor, and classify the index system based on the calculated results, so as to find a solution to the problem. In the process of research, many scholars combined the above two methods, extracted the advantages of both, and proposed the DANP method [43,44]. The method developed rapidly as soon as it was proposed, was widely used in policy evaluation, risk analysis, factor research, and other fields, and became an important method for multicriteria decision-making [45]. In the DANP method, DEMATEL can study the interaction of various factors in a complex system, and convert the relationship between factors into an easy-to-understand system structure model in the form of a matrix or graph. The method replaces the judgment matrix in ANP with the comprehensive influence matrix and combines DEMATEL and ANP to obtain the weight of each factor. The DANP model can not only study the relationship between the influencing factors, it can also study the relationship between dimensions. At the same time, it can improve the degree of fuzzy transformation of data, clarify the logical relationship between the internal effects of each influencing factor, and improve the accuracy and credibility of research results. Therefore, this paper chooses the DANP method to determine the weight. Furthermore, the NK model can be optimized based on the DANP method. Firstly, the traditional NK model makes the K values of all factors equal, that is, all factors are affected by the same amounts of other factors. In fact, factors are affected by different amounts of other factors, and the K values of each factor may be different. Therefore, when the state of one factor changes, it will lead to the change of the state of other factors in different quantities. Secondly, the traditional NK model considers that the weights of all factors are equal. When calculating the system fitness F, the fitness values of all factors are added and then arithmetically averaged, which is consistent with reality. In practice, the contribution of each factor to the system is different, so the weight is not equal [46,47]. In order to overcome the above disadvantages, we modified the NK model with the DANP method to determine the K value and weight of each factor, respectively.

The main contributions of this paper are as follows: (1) A niche influencing factor system of photovoltaic agriculture was constructed from two aspects of resources and functions. (2) The biological evolution NK model was introduced into the study of the niche promotion path, and the NK model was optimized based on the DEMATEL and analytic network process (DANP) method. (3) The optimal path of niche improvement of China’s photovoltaic agriculture was proposed as follows: “technological innovation → policy formulation → resource allocation → economic improvement → social recognition → environmental protection”.

The rest of the paper is organized as follows. The following section constructs the niche influencing factor system of photovoltaic agriculture. In Section 3, methods are presented. Section 4 summarizes the results, and Section 5 discusses them.

## 2. Niche Influencing Factor System Construction

### 2.1. Analytical Framework

**Figure 1:**
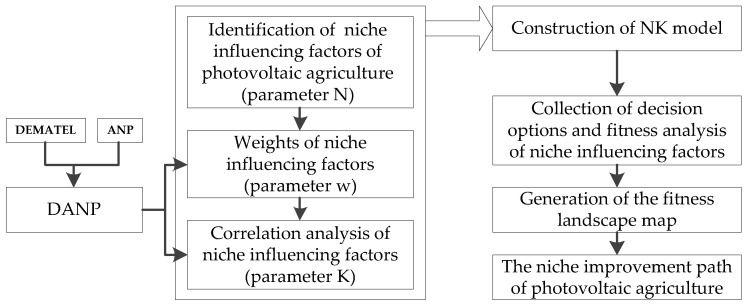
Analytical framework.

To study the niche improvement path of photovoltaic agriculture, this paper firstly identified the niche influencing factors. Then, based on the NK model, this study proposed the method of niche improvement path selection, so as to more accurately explore the complex relationship between the niche influencing factors of photovoltaic agriculture. Further, the impacts of the changes of niche influencing factors of photovoltaic agriculture on the system adaptability were studied. Finally, we explored the optimal path of niche improvement. The analytical framework is shown in Figure 1.

### 2.2. Niche Influencing Factor System Construction of Photovoltaic Agriculture

**Figure 2:**
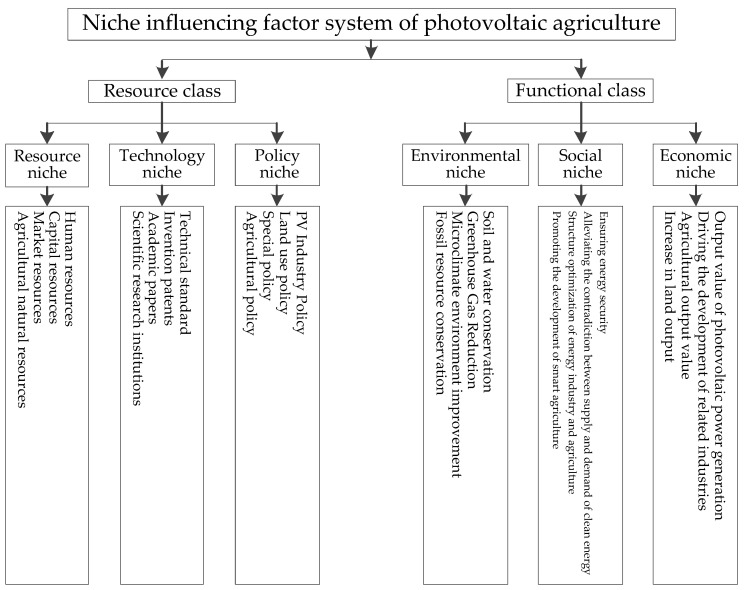
Niche influencing factor system of photovoltaic agriculture.

The ecological niche of photovoltaic agriculture is the functional position of the industrial organization to occupy environmental resources for a certain period and to realize the organization in its natural–economic–social environment. Photovoltaic agriculture occupies a certain number of resources in the dimension of environmental resources, and constructs its own ecological niche, so as to ensure its survival and development. Its resource living space can be divided into technology, policy, and other resources, which include natural resources and other social and economic resources except for technology and policy. After occupying these environmental resources and building the ecological niche, it can play its function and role in the environmental, social, and economic aspects, thus enhancing its status. Therefore, the niche influencing factor system can be conducted from the two aspects of environmental resources and functional state. The former covers resource niche, technology niche, and policy niche, and the latter covers the environmental niche, social niche, and economic niche. The niche influencing factor system of photovoltaic agriculture is shown in Figure 2.

It can be seen from Figure 2 that the influencing factors of the resource niche include agricultural natural resources, market resources, capital resources, and human resources. Among them, agricultural natural resources are the basic resources for the generation and development of photovoltaic agriculture, market resources reflect the society’s demand for photovoltaic agriculture, and capital resources and human resources are the necessary input resources for photovoltaic agriculture development [7]. The influencing factors of the technological niche include scientific research institutions, academic papers, invention patents, and technical standards. Among them, scientific research institutions can greatly promote the development of photovoltaic agriculture [48], academic papers reflect the most cutting-edge innovation achievements in the field of photovoltaic agriculture, invention patents represent the ability to transform productivity, and technical standards can regulate the development of the industry [19]. The influencing factors of the policy niche include agricultural policy, special policy, land use policy, and PV industry policy. Among them, the development of photovoltaic agriculture is deeply affected by the PV industry policy and agricultural policy [7]. The special policy helps photovoltaic agriculture quickly establish its ecological niche, and the land use policy alleviates the land use problem of photovoltaic agriculture. Photovoltaic agriculture can generate photovoltaic electricity, reducing the consumption of fossil resources such as coal, oil, and natural gas, thereby reducing greenhouse gas emissions [49]. The erection of photovoltaic panels hinders the direct exposure of sunlight to crops, thereby changing the microclimate environment, such as light, temperature, and humidity, for crop growth [50]. In some areas with very strong sunlight, the construction of photovoltaic power plants reduces the excessive evaporation of water in the soil, making the originally arid land regain a certain degree of humidity [6]. Therefore, the land has the conditions for plant growth, and green vegetation grows, thereby preventing soil erosion. According to the above analysis, the influencing factors of environmental niche include fossil resource conservation, microclimate environment improvement, greenhouse gas emissions, and soil and water conservation. The development of photovoltaic agriculture promotes the application of photovoltaics in agriculture, thereby promoting the development of smart agriculture. The development of photovoltaic agriculture takes into account the development of photovoltaic industry and agriculture, thereby optimizing the structure of energy industry and agriculture. Photovoltaic agriculture can generate clean electricity, thereby alleviating the contradiction between supply and demand of clean energy [51] and ensuring energy security [52]. Therefore, the influencing factors of social niche include promoting the development of smart agriculture, optimizing the structure of energy industry and agriculture, alleviating the contradiction between supply and demand of clean energy, and ensuring energy security. The development of photovoltaic agriculture improves the efficiency of land use, thereby increasing the output of land [20], which is embodied in the increase in the total output value of agriculture and photovoltaic power generation. In addition, photovoltaic agriculture can also be combined with local industries [40], thereby driving the development of related industries. Based on the above analysis, the influencing factors of the economic niche include increase in land output, agricultural output value, driving the development of related industries, and output value of PV power generation.

## 3. Methods

### 3.1. DANP

The DANP method can be divided into two parts. First, the DEMATEL method is used to construct a network relationship diagram. Secondly, the DEMATEL calculation results are introduced into the DANP method to calculate the index weights.

#### 3.1.1. Construction of Network Relationship Diagram

The first methodological step allows to build the direct influence matrix. Twenty experts in the industry were invited to judge the strength of the correlation between the elements in the niche influencing factor system according to the rules in Table 2. We use the data scored by experts to construct the direct influence matrix M. The influence relationship between each element is represented by 0, 1, 2, 3, and 4. The specific meaning of each value is as follows:

We construct the direct influence matrices of the criterion layer and the network layer. If there are *n* elements in the network layer, we obtain the matrix $M={\left[m_{ij}\right]}_{n\times n}$; $m_{ij}$ is the influence degree of the i-th row element on the j-th column element.

$$M=\left[\begin{array}{ccccc} m_{11} & \cdots & m_{1j} & \cdots & m_{1n}\\ \vdots & & \vdots & & \vdots \\ m_{i1} & \cdots & m_{ij} & \cdots & m_{in}\\ \vdots & & \vdots & & \vdots \\ m_{n1} & \cdots & m_{nj} & \cdots & m_{nn} \end{array}\right]$$

The second methodological step allows to calculate the normalization matrix. We convert the direct influence matrix to a normalized matrix. First, we sum up the elements of each row and column that directly affect the matrix *M*, and obtain the maximum value. We multiply each element in M by the reciprocal of the maximum value to obtain the normalized matrix N, as shown in Formulas (1) and (2).

(1) N=kM

(2) $$k=min\left\{\frac{1}{max_{i}\sum_{j=1}^{n}m_{ij}},\;\frac{1}{max_{j}\sum_{i=1}^{n}m_{ij}}\right\}$$

Here, $max_{i}\overset{n}{\underset{j=1}{\sum }}m_{ij}$ is the maximum value of the row total, and $max_{j}\overset{n}{\underset{i=1}{\sum }}m_{ij}$ is the maximum column total.

The third methodological step allows to calculate the combined impact matrix. After the normalized matrix is obtained, the comprehensive influence matrix can be obtained. The comprehensive influence matrix $X_{D}$ of the criterion layer and the comprehensive influence matrix $X_{C}$ of the network layer can be calculated from their respective normalization matrices according to Formula (3).

(3) $$X=N+N^{2}+N^{3}+\cdots =N{\left(I-N\right)}^{-1}$$

where I is the identity matrix of the same dimension as the normalization matrix.

#### 3.1.2. Weight Calculation

DANP has the advantage of using the DEMATEL method to obtain a comprehensive influence matrix, which replaces a large number of judgment matrices in the ANP method. Therefore, this paper combined the calculation results of DEMATEL with ANP to obtain the weight of each influencing factor.

The fourth methodological step allows to calculate the normalization matrix. This step normalizes the abovementioned comprehensive influence matrix.

The comprehensive influence matrix $X_{D}$ of the criterion layer is standardized by Formulas (4) and (5), that is, the sum of the elements of each row in the matrix is calculated, and then the elements in the row are divided by the sum of elements to obtain the standardized matrix $X_{D}^{\alpha }$.

The standardization method of the comprehensive influence matrix $X_{C}$ of the network layer is different from that of the criterion layer. First, the sub-matrices in the matrix are standardized one by one, and then the standardized matrix $X_{C}^{\alpha }$ is obtained.

Taking the sub-matrix $X_{C}^{\alpha_{23}}$ as an example, the normalization process is shown in Formulas (6)–(8).

(4) $$X_{D}^{\alpha }={\left[x_{D}^{\alpha_{ij}}\right]}_{m\times m}=\left[\begin{array}{ccccc} x_{D}^{11}/d_{1} & \cdots & x_{D}^{1j}/d_{1} & \cdots & x_{D}^{1m}/d_{1}\\ \vdots & & \vdots & & \vdots \\ x_{D}^{i1}/d_{i} & \cdots & x_{D}^{ij}/d_{i} & \cdots & x_{D}^{im}/d_{i}\\ \vdots & & \vdots & & \vdots \\ x_{D}^{m1}/d_{m} & \cdots & x_{D}^{mj}/d_{m} & \cdots & x_{D}^{mm}/d_{m} \end{array}\right]$$

(5) $$d_{i}=\sum_{j=1}^{m}t_{D}^{ij},i=1,2,\cdots ,m$$

(6) $$X_{C}^{\alpha }=\left[\begin{array}{ccccc} X_{C}^{\alpha_{11}} & \cdots & X_{C}^{\alpha_{1j}} & \cdots & X_{C}^{\alpha_{1m}}\\ \vdots & & \vdots & & \vdots \\ X_{C}^{\alpha_{i1}} & \cdots & X_{C}^{\alpha_{ij}} & \cdots & X_{C}^{\alpha_{im}}\\ \vdots & & \vdots & & \vdots \\ X_{C}^{\alpha_{m1}} & \cdots & X_{C}^{\alpha_{mj}} & \cdots & X_{C}^{\alpha_{mm}} \end{array}\right]$$

(7) $$X_{C}^{\alpha_{23}}=\begin{array}{c} C_{21}\\ \vdots \\ C_{2i}\\ \vdots \\ C_{2m_{2}} \end{array}\overset{\begin{array}{ccccc} C_{31} & \cdots & C_{3j} & \cdots & C_{3m_{3}} \end{array}}{\left[\begin{array}{ccccc} x_{11}^{23}/x_{1}^{23} & \cdots & x_{1j}^{23}/x_{1}^{23} & \cdots & x_{1m_{3}}^{23}/x_{1}^{23}\\ \vdots & & \vdots & & \vdots \\ x_{i1}^{23}/x_{i}^{23} & \cdots & x_{ij}^{23}/x_{i}^{23} & \cdots & x_{1m_{3}}^{23}/x_{i}^{23}\\ \vdots & & \vdots & & \vdots \\ x_{m_{21}}^{23}/x_{m_{2}}^{23} & \cdots & x_{m_{2j}}^{23}/x_{m_{2}}^{23} & \cdots & x_{m_{2}m_{3}}^{23}/x_{m_{2}}^{23} \end{array}\right]}$$

(8) $$x_{i}^{23}=\sum_{j=1}^{m_{3}}x_{ij}$$

The fifth methodological step allows to transpose the network layer normalization matrix $X_{C}^{\alpha }$ to obtain a new n×n matrix (the unweighted supermatrix $W^{*}$ obtained by the DANP method). The calculation formula is shown in (9).

(9) $$W^{*}={\left(X_{C}^{\alpha }\right)}^{{}^{\prime }}$$

In the sixth methodological step, it is necessary to multiply each element in the obtained standardization matrix $X_{D}^{\alpha }$ with the corresponding submatrix of the unweighted supermatrix, so as to achieve weighting of each submatrix and finally obtain a weighted supermatrix $W^{\alpha }$. The calculation process is shown in Formula (10).

(10) $$W^{\alpha }=X_{D}^{\alpha }W^{*}=\left[\begin{array}{ccccc} x_{D}^{\alpha_{11}}\times W_{11}^{*} & \cdots & x_{D}^{\alpha_{1i}}\times W_{i1}^{*} & \cdots & x_{D}^{\alpha_{1n}}\times W_{n1}^{*}\\ \vdots & & \vdots & & \vdots \\ x_{D}^{\alpha_{j1}}\times W_{1j}^{*} & & x_{D}^{\alpha_{ji}}\times W_{ij}^{*} & & x_{D}^{\alpha_{jn}}\times W_{nj}^{*}\\ x_{D}^{\alpha_{n1}}\times W_{1n}^{*} & & x_{D}^{\alpha_{ni}}\times W_{in}^{*} & & x_{D}^{\alpha_{nn}}\times W_{nn}^{*} \end{array}\right]$$

where $W_{ij}^{*}$ is the submatrix of $W^{\alpha }$; $x_{D}^{\alpha_{ji}}$ is the element of $X_{D}^{\alpha }$.

Based on the obtained weighted supermatrix $W^{\alpha }$, by exponentiating the matrix $W^{\alpha }$ as shown in Formula (11), the limit supermatrix W with the result convergence and stability can be obtained when the number of powers approaches infinity, and the value corresponding to each element in the matrix is the weight of the element.

(11) $$W=\underset{h\to \infty }{\lim}{\left(W^{\alpha }\right)}^{h}$$

### 3.2. NK Model

If there are N elements in a system, and each element has several alleles, the allele of the element can represent its attribute state, that is, the various attributes that the element may have. The value of this attribute state is not fixed (it can take two states of high and low, or three states of upper, middle, and lower, etc.); alleles are measured by 0, 1, 2, etc. If element i(i=2,…,N) has $A_{i}$ alleles, the number of combinations of attribute states for all elements is

(12) $$A_{1}\times A_{2}\times \cdots \times A_{2}=\overset{N}{\underset{i=1}{\prod }}A_{i}$$

After Kauffman’s many experiments, it was found that if the attribute states of each factor in the system are set to two, the model will be greatly simplified, and the accuracy of the model will not be affected [53]. Therefore, this paper draws on Kauffman’s research conclusions and defines the alleles of each element in the system as two states: 0 and 1. Among them, 0 means that the fitness value of the element is lower than the mean of the fitness of all elements, and 1 means that the fitness value is higher than the mean of the fitness of all elements. Thus, the number of possible combinations of element state attributes is $2^{N}$ [54].

In addition to the number of elements N and allele A, the complexity of the phylogenetic process is also closely related to the parameter K. Kauffman assumes that each factor of the system is affected by the same number of K other factors, then 0≤K≤(N−1). K=0 means that there is no interaction between the factors, that is, the contribution of each factor to the evolution of the system degree is not affected by other factors. K=N−1 indicates that the structure of the system is very complex, and the change of the state of each element will affect the evolution of the system, and each element will also be affected by other elements and indirectly affect the evolution of the system. In the NK model, the change of each element will affect the overall fitness of the system. The complex characteristics of the system itself and the nonlinear relationship between elements make it difficult to determine the fitness function of the entire system. At this time, Kauffman gives a new idea. When an element itself or its related elements change, a random number is drawn from a set of (0,1) uniformly distributed random variables as the element’s contribution to the overall fitness:

(13) $$f_{i}∼U\left(0,1\right),i\in \left\{1,2,\ldots ,N\right\}$$

Based on the above analysis, the system fitness value is the average value of the contribution values of all elements, and then the overall fitness value F of this system is expressed as:

(14) $$F=\frac{1}{N}\overset{N}{\underset{j=1}{\sum }}f_{i}\left(i=1,2,\ldots ,N\right)$$

where $f_{i}$ represents the contribution of the i-th element group to the whole system, and $f_{i}$ is affected by the allele state of itself and related elements at the same time.

**Figure 3:**
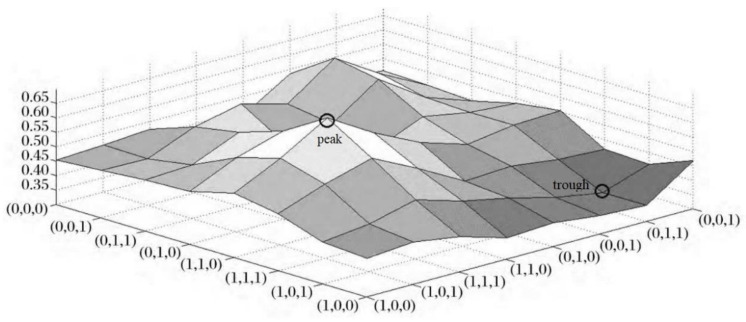
Fitness landscape map.

The NK model is used to continuously simulate and analyze the data. In order to more intuitively describe the relationship between different allele combinations and their fitness values, the data are mapped to a three-dimensional space, which is the fitness landscape map (see Figure 3). In the landscape map, the abscissa is the state combination of a certain part of the allele in the system, and the ordinate is the state combination of another part of the allele. The combination of the abscissa and ordinate data on the bottom surface is the allele combination of all elements, and the height is the fitness value. Since the fitness values of the allele combinations are not equal, there are “peaks” and “troughs” in the fitness landscape. The evolution process of the system is the process of constantly searching for higher points through the change of element alleles, and then climbing from the low point to the highest point on the fitness landscape map. Through the NK model, the number of local optimums in the system can be excavated, and at the same time, the climbing path of the system to reach a higher fitness “peak” can be explored.

There are “peaks” and “troughs” in the fitness landscape, while the NK model pays more attention to “peaks”. Because the NK model combines the fitness landscape theory and uses the “climbing” process on the fitness landscape to show the evolution process of the complex system, the purpose is to explore which main form and structural relationship help the system to achieve a higher fitness value, that is, climbing to a higher “peak” on the fitness landscape. If a “peak” is higher than all other neighboring peaks, that is, the fitness value of this point is higher than that of neighboring points, then the point corresponding to the “peak” is called “local optimum”, and all the fitness landscapes are called “local optimum”. The maximum value of the local optimum is the “global optimum”.

## 4. Results

### 4.1. Determination of the Relationship between Niche Influencing Factors

This paper took resources, technology, policy, environment, society, and economy as the niche influencing factors of photovoltaic agriculture, and abstracted these six factors into six elements in the system for modeling to explore the interaction between them and the impact on niche improvement of photovoltaic agriculture. Then, the optimal niche improvement path of photovoltaic agriculture was explored.

Firstly, the DEMATEL method was used to obtain the comprehensive influence matrix of the first-level influencing factors and the second-level influencing factors (see Table 3 and Table 4).

Secondly, according to the comprehensive influence matrix X, the influence degree, the influenced degree, the centrality degree, and the cause degree of each element of the criterion layer and the network layer are obtained. By summing the elements $x_{ij}$ (i are equal) of a certain row, the influence degree $d_{i}$ of the element i can be calculated, that is, the influence degree of this element on other elements. By summing a certain column of elements $x_{ij}$ (j are equal) in the matrix, the influence degree $r_{j}$ of the j element can be calculated, that is, the degree to which this element is influenced by other elements. By summing the influence degree and the influenced degree of the element, the centrality of the element can be calculated as $c_{i}=d_{i}+r_{i}$, that is, the importance of the element. The higher the centrality, the more significant the effect of this element in the indicator system. If the influence degree of the element is higher than the influenced degree, that is, $d_{i}-r_{i}>0$, it means that the element is the cause element and will affect other elements. If the influence degree is lower than the influenced degree, the difference $d_{i}-r_{i}$ between the two is a negative value, which means that the element is the result element and will be influenced by other elements. The calculated influence degree, affected degree, centrality, and causality of influencing factors at all levels are shown in Table 5.

**Figure 4:**
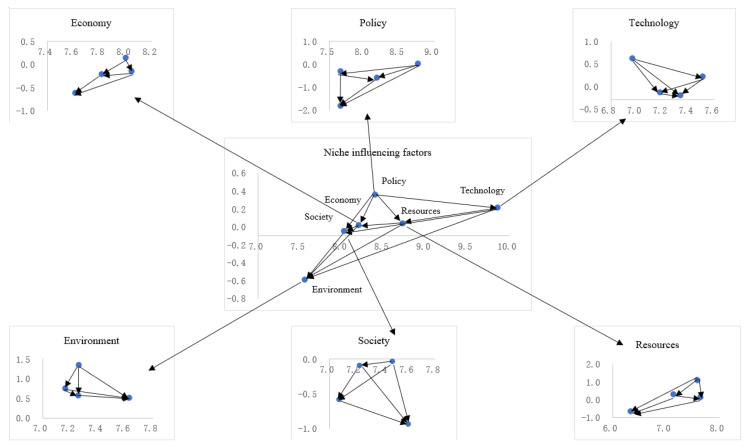
Network relationship diagram of the niche influencing factors.

In order to more intuitively show the importance of each influencing factor in the niche improvement and the influence and affected relationship among factors, the network relationship diagram of the influencing factors of the ecological niche of photovoltaic agriculture was drawn with the centrality as the horizontal axis and the cause degree as the vertical axis (see Figure 4).

Finally, the DANP method was used to calculate the weight of each influencing factor, as shown in Table 6.

According to the cause degree, niche influencing factors can be divided into causal factors and result factors. The positive cause degree means that the factor will affect other factors, which is the cause factor, while the negative cause degree means that the factor is affected by other factors and is the result factor. In the cause degree statistics of the first-level index elements (see Table 5), the cause elements are policy (C_3_), technology (C_2_), resources (C_1_), and economy (C_6_), and the result elements are society (C_5_) and environment (C_4_). The ecological niche of photovoltaic agriculture is mainly affected by the above factors. The ranking of the centrality of the first-level indicators is technology (C_2_), resources (C_1_), policy (C_3_), economy (C_6_), society (C_5_), and environment (C_4_), of which technology (C_2_) is the most critical first-level indicator factor of the niche improvement of photovoltaic agriculture.

It can be seen from Table 6 that the index weights and rankings of the first-level elements are technology C_2_ (0.1902), resources C_1_ (0.1715), economy C_6_ (0.1614), environment C_4_ (0.1600), society C_5_ (0.1591), and policy C_3_ (0.1589). From the above results, it can be seen that technology (C_2_) is the most critical factor affecting the niche improvement of photovoltaic agriculture. Technological progress can not only reduce costs, but also increase land output without negatively impacting the environment, thereby gaining policy support, obtaining more resources, exerting greater economic, social, and environmental functions, and achieving ecological niche enhancement.

### 4.2. NK Model Revision

According to the above DANP method, the relationship and weight of the factors affecting the niche improvement of photovoltaic agriculture were calculated, and then the NK model was revised using the above results. Firstly, we clarify the interaction relationship between the factors in the NK model, and then the value of the parameter K can be obtained. The comprehensive influence matrix calculated by the DEMATEL method can reflect the degree to which each factor is affected by other factors, so this study will calculate the K value of each factor through the comprehensive influence matrix. Because each element of the comprehensive influence factor matrix in Table 3 reflects the degree of influence of row elements on column elements, and the K value reflects the degree of influence of row elements by column elements, the comprehensive influence matrix is first transposed, as shown in Table 7.

Then, it is necessary to determine the influence relationship between the factors, and before this, the threshold value must be set first. There is no uniform standard for the threshold value. Some studies use the method of expert discussion to obtain it, and some studies set the threshold value as the average value of the influence of all elements. In this study, the former was adopted, and 0.5 was used as the threshold. When the influence degree of the elements in the matrix is greater than 0.5, the value is 1; otherwise, the value is 0. The matrix in Table 7 was changed to an adjacency matrix (see Table 8).

Based on this, the influence matrix of the revised NK model can be obtained, which can reflect the influence relationship of each element, as shown in Table 9.

It can be seen from the above table that after using the DEMATEL method to revise the NK model, the K value of each element is exactly 5. It is explained that these six elements will affect the other five elements, and are also affected by the other five elements.

Furthermore, this paper will revise the formula for calculating the overall fitness value of the system in the NK model. The fitness of influencing factors is resource $f_{1}$, technology $f_{2}$, policy $f_{3}$, environment $f_{4}$, society $f_{5}$, and economy $f_{6}$.

(15) $$F=\frac{1}{6}\sum_{i=1}^{6}f_{i},i=1,2,\ldots ,6$$

Formula (15) defaults to the same importance of the six influencing factors, and the calculation results of the DANP method show that each factor does not have the same weight. Based on the size of the weight, if the path direction is guided to a factor with a larger weight in the early path selection, then when the NK model explores the path, it can obtain a higher fitness value in less time [55]. Therefore, this study introduced the weights of six influencing factors, namely, resources (0.1715), technology (0.1902), policy (0.1589), environment (0.1600), society (0.1591), and economy (0.1614), into the NK model and Formula (15), and a new fitness calculation Formula (16) was obtained.

(16) $$F=\sum_{i=1}^{N}w_{i}f_{i}=(0.1715f_{1}+0.1902f_{2}+0.1589f_{3}+0.16f_{4}+0.1591f_{5}+0.1614f_{6})$$

### 4.3. MATLAB Simulation

After revising the NK model, the simulation technology will be used to simulate the influence of the changes and interactions of influencing factors on the overall fitness of the ecological niche of photovoltaic agriculture, compare the fitness values in the terrain, find the maximum value, and set it as the “global optimum point”. The climbing path can be displayed through the above process, and explains how the states of niche influencing factors of photovoltaic agriculture change, so as to obtain an improvement path of the ecological niche. This study used MATLAB to code and simulate the change process of niche influencing factors in the NK model, and assigned random values to each influencing factor as the fitness. Finally, Formula (16) was used to calculate the overall fitness value of the system.

There are six niche influencing factors in the NK model constructed in this paper. As mentioned above, the system fitness value is not greatly affected by the number of the states of influencing factors, so here we set 0 and 1 as the two states of all niche influencing factors. In this way, there are 64 states in the whole system, including {0,0,0,0,0,0}, {1,0,0,0,0,0}, {0,1,0,0,0,0}, {0,0,1,0,0,0}, {0,0,0,1,0,0}, {0,0,0,0,1,0}, etc. Considering the complexity of the ecological niche of photovoltaic agriculture, this study limits the number of influencing factors for each state change to one without loss of generality.

It can be seen from the above that once the state of each niche influencing factor is determined, the corresponding fitness value can be obtained. The exploration of the best path for niche improvement is the search for higher fitness values. From this aspect, the process of exploring the best path is the evolution process of various niche influencing factors, and this process finally shows up as influencing factors climb the landscape map by constantly changing states. During this process, when the state of one factor changes, a random, new value will be assigned to the factor. At this time, other factors affected by this factor will also update the random assignment, and the overall fitness value of the system will change accordingly. When the fitness value of the system is greater than the fitness value before the state change, the change is an effective change, and the change of the factor state can be retained. On the contrary, if the system fitness value after the factor state change is smaller than the original value, then the change of the factor state shall be discarded.

MATLAB was used to calculate the fitness values of niche influencing factors of photovoltaic agriculture in different states (see Table 10). In order to ensure the reliability and stability of the simulation results, a total of 100,000 simulations were carried out in this study (the results of many studies show that stable and reliable results can be obtained when the simulation reaches a certain number of times, such as more than 50,000 times). Generally speaking, the initial state of each factor is 0; at this time, the state of the system is {0,0,0,0,0,0}, and its fitness value is 0.3484. Then, the states of influencing factors are changed, and only one factor state is changed at a time until the states of all factors are 1, and the system state at this time is {1,1,1,1,1,1}. As mentioned above, we first consider the change of the state of one factor, changing it from “0” to “1”; at this time, the state of the system can be one of {1,0,0,0,0,0}, {0,1,0,0,0,0}, {0,0,1,0,0,0}, {0,0,0,1,0,0}, {0,0,0,0,1,0}, or {0,0,0,0,0,1}. Statistical analysis of the simulation results in the above six states shows that technological innovation as the first state change factor accounts for the highest proportion of the results, which is 17.9%. Therefore, the improvement of technology should be regarded as the first key step in the niche improvement process of photovoltaic agriculture. The state at this time becomes {0,1,0,0,0,0}, and the fitness value is 0.4068. On the basis of the state {0,1,0,0,0,0}, we continue to change the states of other influencing factors, including {1,1,0,0,0,0}, {0,1,1,0,0,0}, {0,1,0,1,0,0}, {0,1,0,0,1,0}, and {0,1,0,0,0,1}. Statistical analysis of the simulation results of the above five states shows that the number of reservations of state {0,1,1,0,0,0} exceeds that of other states, accounting for 22.7% of the total. This state change corresponds to the state change of the policy influencing factor, indicating that after technological innovation, the second key step in the niche improvement path of photovoltaic agriculture should be policy formulation. By repeating the above method, it can be determined that the sequence of key element combinations for increasing the fitness value of photovoltaic agriculture is as follows: {0,0,0,0,0,0} → {0,1,0,0,0,0} → {0,1,1,0,0,0} → {1,1,1,0,0,0} → {1,1,1,0,0,1} → {1,1,1,1,0,1} → {1,1,1,1,1,1}, and the fitness values are 0.3484, 0.4068, 0.4558, 0.4782, 0.7153, 0.7315, and 0.7665. China’s photovoltaic agriculture should follow the path of technological innovation → policy formulation → resource allocation → economic improvement → social recognition → and environmental protection in order to improve the ecological niche.

**Figure 5:**
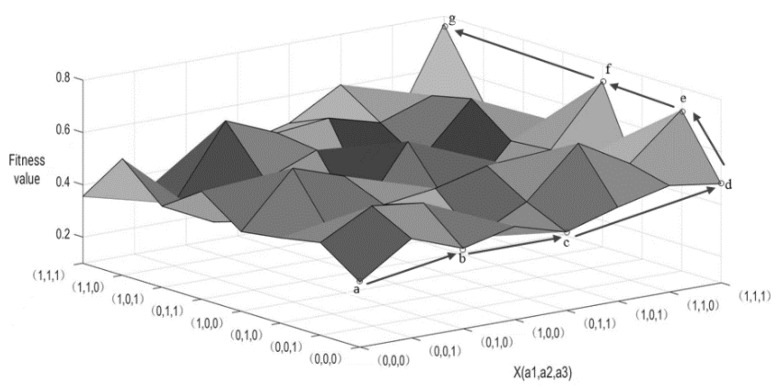
Fitness landscape of the ecological niche of photovoltaic agriculture.

Based on the above analysis results, a schematic diagram of the fitness landscape and climbing process of the ecological niche of photovoltaic agriculture was constructed (see Figure 5). The process of exploring the improvement path of the ecological niche of photovoltaic agriculture is the process of seeking various combinations of key factors to improve the niche fitness value. As the states of alleles of key factors change from “0” to “1”, the overall fitness value of the system can be improved, thus reflecting the “climbing” process on the fitness landscape map.

Each vertex in Figure 5 represents a different combination of the niche states of photovoltaic agriculture. The arrows in the landscape indicate the evolution path of photovoltaic agriculture from lower niche fitness combination to higher niche fitness combination, and the solid arrows indicate the optimal path of photovoltaic agriculture from lower niche fitness to higher niche fitness, that is, the niche improvement path of photovoltaic agriculture explored in this paper. As can be seen from the figure, taking a as the starting point, by analyzing the fitness values of the surrounding points, passing through points b, c, d, e, and f in turn, the process of finally reaching the global optimal point, g point, is the search process of niche improvement path of photovoltaic agriculture. The state combinations of each influencing factor corresponding to each point are {0,0,0,0,0,0}, {0,1,0,0,0,0}, {0,1,1,0,0,0}, {1,1,1,0,0,0}, {1,1,1,0,0,1}, {1,1,1,1,0,1}, and {1,1,1,1,1,1}, and the specific niche improvement path of photovoltaic agriculture is obtained as follows: a→b→c→d→e→f→g, namely, “technological innovation → policy formulation → resource allocation → economic improvement → social recognition → environmental protection”.

## 5. Discussion

Through the analysis of the NK model and fitness landscape, we can judge for which situation the niche factor combination order has a high niche fitness value, and the continuous evolution process according to the combination order is the path of niche promotion of photovoltaic agriculture.

First of all, there are many combination points in the niche fitness landscape map, where the highest niche combination state is {1,1,1,1,1,1}. At this point, the system reached the highest fitness value, and also realized the optimal niche state, which shows that the reasonable configuration of niche factors can make photovoltaic agriculture obtain the optimal niche.

Secondly, the path to improve the ecological niche level can be explored through the niche fitness landscape map. For example, when the niche combination state of photovoltaic agriculture is {1,1,1,0,0,1}, the niche fitness value is 0.7153, and there are two paths to choose from. Path 1 is {1,1,1,0,0,1} → {1,1,1,1,0,1} → {1,1,1,1,1,1,1}, emphasizing the strengthening of environmental niche factors first, and then the strengthening of social niche factors. The fitness value increases from 0.7153 to 0.3641. This path will reduce the niche fitness value and is an invalid path, so it should be discarded. Path 2 is {1,1,1,0,0,1} → {1,1,1,0,1,1} → {1,1,1,1,1,1}, emphasizing the strengthening of social niche factors first, and then the strengthening of environmental niche factors. The fitness value increases from 0.7153 to 0.7315 and finally reaches 0.7665. This path can promote the gradual improvement of the niche fitness value and is an effective path that can be selected.

Thirdly, the accumulation and dependence relationship of niche factors can be found through the niche fitness landscape. In the fitness landscape, there are multiple paths from the point {0,0,0,0,0,0} to the highest point {1,1,1,1,1,1}, but only the path of {0,0,0,0,0,0} → {0,1,0,0,0,0} → {0,1,1,0,0,0} → {1,1,1,0,0,0} → {1,1,1,0,0,1} → {1,1,1,1,0,1} → {1,1,1,1,1,1} can make its ecological niche have the highest local fitness value in the whole evolution process. At the same time, this path is also the optimal path to promote the gradual improvement of the niche fitness value, and this optimal path also reflects the gradual accumulation process among the various niche factors.

Finally, photovoltaic agriculture should choose a reasonable niche improvement path according to the niche state. When the niche fitness value is at a low level, the ecological niche should be improved through technological innovation or policy support to lay a foundation for the next stage of development. The specific path analysis is as follows:

(1) Technology

Technology plays a crucial role in searching for the highest point in the niche fitness landscape of photovoltaic agriculture. Therefore, the path of niche improvement should first consider strengthening technological innovation and improving the utilization efficiency of technology on resources. As far as the generation of photovoltaic agriculture is concerned, technological progress is crucial. The progress of photovoltaic technology has contributed to the formation of the photovoltaic industry. The reduction of photovoltaic power generation cost and the environmental friendliness of the photovoltaic technology itself makes it have a lot of application space in agriculture. The development of the photovoltaic industry needs more resource support, and the development of the “symbiotic” technology [56] of photovoltaic power generation and agricultural production makes it possible to form the dual-use land system. The above two aspects constitute photovoltaic agriculture. With the further development of photovoltaic technology and the “symbiotic” technology, photovoltaic agriculture, relying on its basic ecological niche, will play its economic, social, and environmental functions to a greater extent, and realize the improvement of the ecological niche. Therefore, in the process of the formation and development of photovoltaic agriculture, technology is in the primary position.

(2) Policy

In the early stages of the development of innovative technologies and new things, policies can play a necessary supporting role to help them establish a niche. At present, China has issued a number of policies related to photovoltaic agriculture, for example, the *Notice on Further Implementing Relevant Policies on Distributed Photovoltaic Power Generation* released by the National Energy Administration of China, the *13th Five-Year Plan for Solar Energy Development* issued by the National Energy Administration of China, and the *Intelligent Photovoltaic Industry Development Action Plan (2018–2020)* issued by Ministry of Industry and Information Technology Department of China. These policies mention photovoltaic agriculture, which has played a role in promoting the development of photovoltaic agriculture. Therefore, the policy can play a necessary role in supporting the niche establishment of photovoltaic agriculture.

(3) Resources

Resources are a necessary factor for photovoltaic agriculture to realize its economic, social, and environmental functions. In addition to natural resources, the resources in this paper also include market, capital, and human resources. Resource allocation is affected by policies, whose support for innovation can be reflected in the allocation of various resources needed for their development [57]. Specifically for the development of photovoltaic agriculture, land resources are the necessary resources for project construction. Whether the local agricultural and forestry departments can approve the construction land of photovoltaic agriculture projects within the scope of the policy is very important to the development of photovoltaic agriculture. Similarly, whether the energy management department can divide a certain proportion of the photovoltaic market specifically for the development of photovoltaic agriculture, and whether the agriculture and the photovoltaic industry can invest a certain amount of funds and human resources to support the development of photovoltaic agriculture, also affect the development of photovoltaic agriculture, thus affecting the ecological niche of photovoltaic agriculture.

(4) Economy

After the above various resource factors have been possessed, economic factors have become the primary factor for the realization of the function of photovoltaic agriculture. The investment and construction of photovoltaic agriculture projects and the operation of photovoltaic agriculture enterprises are carried out by all stakeholders through a certain interest connection mechanism. The land owner, the project owner, and the operator should realize their economic interests through the project operation or the enterprise operation. Otherwise, the project will not be able to operate normally, the enterprises will not be able to operate normally, and the social and environmental functions of photovoltaic agriculture will be out of the question.

(5) Society

After the realization of economic functions, the realization of social functions has a better foundation. Based on the normal operation of the project and the enterprise, photovoltaic agriculture can provide jobs. As photovoltaic agriculture has produced some output in photovoltaic power and agriculture, it has realized its social functions such as meeting the demands for clean energy capacity, energy security, and food security. The realization of these social functions further increases its social recognition and contributes to the promotion of photovoltaic agriculture and the realization of its various functions.

(6) Environment

For photovoltaic agriculture, the environment is both the cause and the result of the development of this technology or approach. As mentioned above, the generation and development of photovoltaic agriculture is mainly due to the development of photovoltaic technology and the photovoltaic industry, and the development of photovoltaic agriculture is largely due to environmental protection. Because of this, the photovoltaic power generation technology has the advantage of shifting to agricultural production, and the photovoltaic industry also has the possibility of an “invasion” to agricultural land resources. With the integration of photovoltaics and agriculture, it can not only improve the microclimate environment of agricultural production but also play an unexpected environmental protection effect under some extreme natural conditions. For example, in the construction of photovoltaic power stations in arid, semiarid, or desert areas, the blocking of photovoltaic panels from sunlight reduces the evaporation of water in the land, making the land vibrant and suitable for growing green plants, thus playing a role in preventing soil erosion and realizing environmental protection.

The results of this paper are basically consistent with those of previous studies. For the sustainable development of China’s photovoltaic agriculture, many studies first proposed technical suggestions such as the establishment of unified technical standards [49] and the technological cooperation between photovoltaic power generation and agricultural production [40]. Then, policy support is necessary. It helps establish an incentive mechanism [58] and improve the enthusiasm of participants [25]. On the basis of the above two aspects, photovoltaic agriculture can better play its economic effect and gain social recognition [30]. Finally, the purpose of environmental protection will be realized [27,59], and photovoltaic agriculture will achieve sustainable development. However, the existing literature does not mention resource allocation. This paper proposes this point in particular, and regards it as the third step of the niche improvement path of photovoltaic agriculture. We also integrated the above factors, and simulated the niche improvement path of photovoltaic agriculture based on this. This is a more comprehensive and objective result based on the NK model optimized by DANP.

## 6. Conclusions

This paper first constructed the niche influencing factor system of photovoltaic agriculture. Then, the NK model was modified by DANP method. Finally, based on the constructed niche influencing factor system and the optimized NK model, the optimal niche improvement path of photovoltaic agriculture was explored. We found that the niche influencing factor system of photovoltaic agriculture includes six aspects: resource niche, technology niche, policy niche, environmental niche, social niche, economic niche. The introduction of DANP method into NK model can overcome the shortcomings of traditional NK model, and the results are more objective. The interaction between the above six niche influencing factors determines the niche fitness of photovoltaic agriculture, and the change of the niche fitness and the path of niche improvement are synergistic. The optimal niche improvement path of photovoltaic agriculture in China is “technological innovation → policy formulation → resource allocation → economic improvement → social recognition → environmental protection”. In summary, China’s photovoltaic agriculture should correctly position its own resource advantages, industrial advantages, and social needs, and combine these with the actual situation and its own unique ecological niche to seek the correct development path. At the same time, we should pay attention to the development and improvement of the ecological niche. Only by continuously expanding its own ecological niche can we promote the sustainable development of photovoltaic agriculture.

This paper studied the niche improvement path of photovoltaic agriculture based on the basic concept of niche in niche theory, but there are some concepts in niche theory, such as niche width, niche overlap, etc., which are not included in this study due to the limited scope of the study. The main research data of this paper were obtained through expert survey. Although the questionnaires were carefully designed, distributed, and retrieved according to the research needs, the sample size is relatively small. In this paper, corresponding models were established according to the needs in the research process. These models have good accuracy, but in terms of their scope of use and effect, they still need to be further tested. Future research can be improved from the above aspects.

## Figures and Tables

**Table 1:** Development history of photovoltaics application in agriculture.

Year/Period	Landmark Events/Development Status
1975	The first photovoltaic water pump was launched.
Early 1980s	The Lewis Research Center of NASA launched a global market forecasting study on the use of photovoltaic systems in the agricultural sector.
1990s	The application of photovoltaics in agriculture encountered many obstacles, which mainly came from the economic, institutional, and social aspects.
After 2000	The application of photovoltaics in agriculture makes good progress, and reflects the trend of diversification.

**Table 2:** Score table of influence relationship.

Score	Meaning (Row Element to Column Element)
0	No effect
1	Very weak effect
2	Weak effect
3	Strong effect
4	Very strong effect

**Table 3:** Comprehensive influence matrix of the first-level influencing factors.

	C_1_	C_2_	C_3_	C_4_	C_5_	C_6_
C_1_	0.6303	0.8910	0.7311	0.6941	0.6914	0.7464
C_2_	0.9107	0.7964	0.7751	0.8645	0.8166	0.8724
C_3_	0.7795	0.8895	0.5757	0.7000	0.7328	0.7008
C_4_	0.6480	0.6595	0.5663	0.4651	0.5664	0.5752
C_5_	0.6783	0.7766	0.6282	0.7336	0.5274	0.6423
C_6_	0.6938	0.8070	0.7377	0.6146	0.7007	0.5538

**Table 4:** Comprehensive influence matrix of the second-level influencing factors.

	C_11_	C_12_	C_13_	C_14_	C_21_	C_22_	C_23_	C_24_	C_31_	C_32_	C_33_	C_34_	C_41_	C_42_	C_43_	C_44_	C_51_	C_52_	C_53_	C_54_	C_61_	C_62_	C_63_	C_64_
C_11_	0.107	0.128	0.158	0.127	0.176	0.202	0.179	0.178	0.147	0.158	0.153	0.155	0.152	0.157	0.172	0.167	0.121	0.134	0.116	0.140	0.174	0.176	0.170	0.166
C_12_	0.150	0.122	0.179	0.171	0.188	0.228	0.215	0.214	0.205	0.204	0.197	0.200	0.169	0.176	0.191	0.185	0.136	0.140	0.136	0.161	0.194	0.200	0.190	0.185
C_13_	0.126	0.145	0.129	0.169	0.162	0.210	0.198	0.186	0.189	0.188	0.182	0.185	0.144	0.161	0.164	0.170	0.125	0.138	0.123	0.135	0.143	0.182	0.163	0.170
C_14_	0.118	0.117	0.142	0.090	0.147	0.167	0.157	0.156	0.115	0.127	0.112	0.125	0.092	0.105	0.120	0.105	0.099	0.108	0.104	0.101	0.109	0.119	0.107	0.103
C_21_	0.119	0.139	0.158	0.152	0.128	0.201	0.189	0.177	0.170	0.169	0.164	0.166	0.138	0.166	0.159	0.153	0.121	0.145	0.129	0.141	0.160	0.162	0.145	0.129
C_22_	0.109	0.106	0.121	0.115	0.114	0.121	0.125	0.126	0.130	0.130	0.125	0.127	0.095	0.142	0.134	0.118	0.100	0.111	0.107	0.128	0.146	0.146	0.133	0.118
C_23_	0.147	0.144	0.163	0.156	0.181	0.206	0.147	0.194	0.175	0.174	0.168	0.158	0.131	0.159	0.152	0.147	0.135	0.148	0.132	0.155	0.165	0.167	0.150	0.157
C_24_	0.176	0.172	0.194	0.185	0.190	0.231	0.206	0.170	0.207	0.206	0.200	0.190	0.148	0.178	0.172	0.177	0.138	0.165	0.160	0.174	0.196	0.213	0.182	0.176
C_31_	0.136	0.133	0.163	0.157	0.182	0.196	0.195	0.183	0.189	0.187	0.181	0.133	0.130	0.147	0.152	0.134	0.124	0.137	0.156	0.167	0.153	0.191	0.150	0.145
C_32_	0.162	0.148	0.167	0.172	0.186	0.213	0.188	0.187	0.190	0.142	0.172	0.186	0.146	0.163	0.145	0.151	0.115	0.140	0.159	0.160	0.157	0.195	0.167	0.137
C_33_	0.155	0.140	0.159	0.163	0.180	0.217	0.204	0.204	0.195	0.194	0.141	0.190	0.138	0.155	0.160	0.154	0.129	0.144	0.163	0.164	0.173	0.189	0.182	0.176
C_34_	0.114	0.124	0.140	0.146	0.169	0.193	0.182	0.181	0.125	0.149	0.145	0.159	0.120	0.137	0.141	0.148	0.103	0.116	0.146	0.157	0.142	0.178	0.151	0.122
C_41_	0.134	0.131	0.149	0.142	0.168	0.205	0.181	0.170	0.160	0.172	0.167	0.182	0.105	0.158	0.163	0.146	0.146	0.159	0.144	0.144	0.164	0.190	0.149	0.155
C_42_	0.137	0.122	0.164	0.146	0.148	0.197	0.184	0.196	0.188	0.187	0.170	0.184	0.154	0.124	0.165	0.148	0.137	0.162	0.136	0.158	0.177	0.182	0.152	0.147
C_43_	0.117	0.114	0.143	0.137	0.149	0.196	0.185	0.174	0.166	0.165	0.171	0.162	0.122	0.151	0.120	0.139	0.141	0.142	0.139	0.138	0.157	0.170	0.142	0.137
C_44_	0.127	0.112	0.152	0.135	0.160	0.172	0.173	0.161	0.164	0.163	0.158	0.160	0.122	0.137	0.154	0.113	0.128	0.140	0.124	0.158	0.131	0.180	0.152	0.148
C_51_	0.161	0.158	0.179	0.171	0.187	0.227	0.213	0.213	0.191	0.202	0.185	0.199	0.132	0.151	0.191	0.186	0.112	0.162	0.169	0.171	0.180	0.209	0.178	0.172
C_52_	0.149	0.135	0.165	0.158	0.173	0.199	0.176	0.198	0.177	0.166	0.171	0.186	0.133	0.149	0.179	0.173	0.150	0.115	0.136	0.159	0.167	0.172	0.165	0.160
C_53_	0.151	0.147	0.167	0.160	0.175	0.201	0.178	0.177	0.180	0.179	0.185	0.176	0.122	0.151	0.181	0.175	0.150	0.152	0.112	0.161	0.169	0.196	0.168	0.150
C_54_	0.155	0.151	0.171	0.175	0.168	0.206	0.193	0.193	0.196	0.183	0.177	0.167	0.138	0.155	0.184	0.178	0.143	0.167	0.139	0.128	0.161	0.189	0.182	0.177
C_61_	0.130	0.115	0.143	0.113	0.126	0.148	0.149	0.161	0.143	0.142	0.137	0.139	0.128	0.152	0.145	0.140	0.086	0.120	0.126	0.137	0.110	0.159	0.143	0.152
C_62_	0.121	0.118	0.146	0.129	0.152	0.186	0.175	0.176	0.146	0.156	0.151	0.141	0.106	0.156	0.146	0.130	0.087	0.123	0.106	0.129	0.149	0.125	0.146	0.130
C_63_	0.144	0.141	0.148	0.140	0.178	0.202	0.190	0.190	0.171	0.159	0.153	0.154	0.141	0.146	0.149	0.155	0.109	0.146	0.117	0.153	0.174	0.176	0.123	0.167
C_64_	0.139	0.125	0.142	0.124	0.173	0.185	0.174	0.184	0.177	0.177	0.148	0.161	0.148	0.152	0.155	0.138	0.116	0.118	0.114	0.125	0.157	0.182	0.142	0.113

**Table 5:** Centrality and causality of influencing factors.

First-Level Factors	$d_{i}$ (Ranking)	$r_{i}$ (Ranking)	$d_{i}+r_{i}$ (Ranking)	$d_{i}-r_{i}$ (Ranking)	Second-Level Factors	$d_{i}$ (Ranking)	$r_{i}$ (Ranking)	$d_{i}+r_{i}$ (Ranking)	$d_{i}-r_{i}$ (Ranking)
Resources C_1_	4.3844/ (2)	4.3406/ (2)	8.7250/ (2)	0.0437/ (3)	Agricultural natural resources C_11_	2.8470/(24)	3.4950/(18)	6.3420/(24)	−0.6480/(22)
					Market resources C_12_	3.7175/(15)	3.4243/(19)	7.1418/(21)	0.2932/(7)
					Capital resources C_13_	4.3372/(2)	3.2533/(21)	7.5905/(12)	1.0838/(2)
					Human resources C_14_	3.8858/(9)	3.7610/(12)	7.6468/(8)	0.1247/(10)
Technology C_2_	5.0356/ (1)	4.8201/ (1)	9.8557/ (1)	0.2156/ (2)	Scientific research institutions C_21_	3.7926/(13)	3.1753/(23)	6.9679/(23)	0.6173/(4)
					Academic papers C_22_	3.8634/(10)	3.6491/(16)	7.5125/(13)	0.2144/(8)
					Invention patents C_23_	3.5739/(17)	3.7643/(11)	7.3382/(15)	−0.1904/(16)
					Technical standards C_24_	3.5241/(19)	3.6552/(15)	7.1793/(19)	−0.1311/(14)
Policy C_3_	4.3783/ (3)	4.0141/ (6)	8.3924/ (3)	0.3642/ (1)	Agricultural policy C_31_	3.6779/(16)	3.9836/(8)	7.6616/(6)	−0.3057/(18)
					Special policy C_32_	2.9249/(23)	4.7336/(1)	7.6585/(7)	−1.8087/(24)
					Land use policy C_33_	3.8047/(11)	4.3768/(2)	8.1815/(2)	−0.5722/(19)
					PV industry policy C_34_	4.3979/(1)	4.3752/(3)	8.7731/(1)	0.0228/(11)
Environment C_4_	3.4805/ (6)	4.0719/ (4)	7.5524/ (6)	−0.5913/ (6)	Fossil resource conservation C_41_	4.3006/(3)	2.9620/(24)	7.2627/(16)	1.3386/(1)
					Microclimate environment improvement C_42_	3.9150/(8)	3.3448/(20)	7.2599/(17)	0.5702/(5)
					Greenhouse gas reduction C_43_	3.9628/(6)	3.2053/(22)	7.1681/(20)	0.7576/(3)
					Soil and water conservation C_44_	4.0666/(5)	3.5627/(17)	7.6293/(9)	0.5039/(6)
Society C_5_	3.9864/ (5)	4.0353/ (5)	8.0217/ (5)	−0.0489/ (5)	Promoting the development of smart agriculture C_51_	3.2447/(22)	3.8308/(10)	7.0754/(22)	−0.5861/(20)
					Structure optimization of energy industry and agriculture C_52_	3.3286/(21)	4.2710/(4)	7.5996/(11)	−0.9424/(23)
					Alleviating the contradiction between supply and demand of clean energy C_53_	3.7190/(14)	3.7589/(13)	7.4780/(14)	−0.0399/(12)
					Ensuring energy security C_54_	3.5657/(18)	3.6656/(14)	7.2313/(18)	−0.0999/(13)
Economy C_6_	4.1076/ (4)	4.0909/ (3)	8.1984/ (4)	0.0167/ (4)	Increase in land output C_61_	3.4970/(20)	4.1149/(5)	7.6119/(10)	−0.6179/(21)
					Agricultural output value C_62_	3.9464/(7)	4.0964/(6)	8.0428/(3)	−0.1501/(15)
					Driving the development of related industries C_63_	4.0682/(4)	3.9292/(9)	7.9974/(4)	0.1390/(9)
					Output value of PV power generation C_64_	3.8044/(12)	4.0109/(7)	7.8153/(5)	−0.2065/(17)

**Table 6:** Weights of factors affecting the niche improvement of photovoltaic agriculture.

Dimension	Weight of First-Level Indicator	Ranking	Influencing Factor	Weight of Second-Level Indicator	Ranking
Resources C_1_	0.1715	2	Agricultural natural resources C_11_	0.0440	5
			Market resources C_12_	0.0410	11
			Capital resources C_13_	0.0397	17
			Human resources C_14_	0.0468	4
Technology C_2_	0.1902	1	Scientific research institutions C_21_	0.0422	9
			Academic papers C_22_	0.0488	2
			Invention patents C_23_	0.0508	1
			Technical standards C_24_	0.0485	3
Policy C_3_	0.1589	6	Agricultural policy C_31_	0.0362	24
			Special policy C_32_	0.0429	8
			Land use policy C_33_	0.0399	15
			PV industry policy C_34_	0.0398	16
Environment C_4_	0.1600	4	Fossil resource conservation C_41_	0.0362	23
			Microclimate environment improvement C_42_	0.0411	10
			Greenhouse gas reduction C_43_	0.0391	20
			Soil and water conservation C_44_	0.0436	7
Society C_5_	0.1591	5	Promoting the development of smart agriculture C_51_	0.0394	18
			Structure optimization of energy industry and agriculture C_52_	0.0440	6
			Alleviating the contradiction between supply and demand of clean energy C_53_	0.0387	21
			Ensuring energy security C_54_	0.0371	22
Economy C_6_	0.1614	3	Increase in land output C_61_	0.0410	12
			Agricultural output value C_62_	0.0410	13
			Driving the development of related industries C_63_	0.0393	19
			Output value of PV power generation C_64_	0.0401	14

**Table 7:** Transpose matrix of comprehensive influence matrix of first-level influencing factors.

	C_1_	C_2_	C_3_	C_4_	C_5_	C_6_
C_1_	0.6303	0.9107	0.7795	0.6480	0.6783	0.6938
C_2_	0.8910	0.7964	0.8895	0.6595	0.7766	0.8070
C_3_	0.7311	0.7751	0.5757	0.5663	0.6282	0.7377
C_4_	0.6941	0.8645	0.7000	0.4651	0.7336	0.6146
C_5_	0.6914	0.8166	0.7328	0.5664	0.5274	0.7007
C_6_	0.7464	0.8725	0.7008	0.57526	0.6423	0.5538

**Table 8:** Adjacency matrix of influencing factors.

	C_1_	C_2_	C_3_	C_4_	C_5_	C_6_
C_1_	1	1	1	1	1	1
C_2_	1	1	1	1	1	1
C_3_	1	1	1	1	1	1
C_4_	1	1	1	0	1	1
C_5_	1	1	1	1	1	1
C_6_	1	1	1	1	1	1

**Table 9:** Influence matrix of the revised NK model.

	C_1_	C_2_	C_3_	C_4_	C_5_	C_6_
C_1_	1	1	1	1	1	1
C_2_	1	1	1	1	1	1
C_3_	1	1	1	1	1	1
C_4_	1	1	1	1	1	1
C_5_	1	1	1	1	1	1
C_6_	1	1	1	1	1	1

**Table 10:** System fitness value changes in different states.

a_1_	a_2_	a_3_	a_4_	a_5_	a_6_	f_1_	f_2_	f_3_	f_4_	f_5_	f_6_	F	
0	0	0	0	0	0	0.1795	0.5061	0.3155	0.5533	0.0858	0.4234	0.3484	(a)
0	0	0	0	0	1	0.0545	0.9356	0.0158	0.6869	0.5364	0.4805	0.4535	
0	0	0	0	1	0	0.3275	0.7958	0.9265	0.5536	0.2429	0.9375	0.4255	
0	0	0	1	0	0	0.4291	0.2116	0.4754	0.4327	0.5992	0.9427	0.5121	
0	0	1	0	0	0	0.0781	0.3601	0.8208	0.6155	0.0893	0.9105	0.4793	
0	1	0	0	0	0	0.1473	0.6082	0.4251	0.1568	0.6492	0.5189	0.4068	(b)
1	0	0	0	0	0	0.2215	0.1160	0.1163	0.2906	0.8182	0.8670	0.3993	
0	0	0	0	1	1	0.1010	0.7160	0.3048	0.2630	0.6818	0.4035	0.4036	
0	0	0	1	0	1	0.0574	0.9941	0.0217	0.3584	0.7311	0.3320	0.4093	
0	0	0	1	1	0	0.6498	0.7502	0.5553	0.2836	0.7363	0.3355	0.5454	
0	0	1	0	0	1	0.9268	0.1584	0.9942	0.1349	0.7546	0.4258	0.5587	
0	0	1	0	1	0	0.5632	0.6834	0.0858	0.3070	0.4229	0.3287	0.3974	
0	0	1	1	0	0	0.3752	0.0820	0.7039	0.1062	0.6763	0.2666	0.3618	
0	1	0	0	0	1	0.4580	0.9125	0.9927	0.6890	0.9513	0.4272	0.5350	
0	1	0	0	1	0	0.5254	0.0256	0.4689	0.2532	0.8073	0.8507	0.4825	
0	1	0	1	0	0	0.6176	0.5867	0.3789	0.7606	0.3402	0.2008	0.4914	
1	0	0	0	0	1	0.7901	0.4045	0.8339	0.7678	0.4881	0.6955	0.4691	
1	0	0	0	1	0	0.4368	0.5725	0.4403	0.9266	0.5518	0.3731	0.5608	
1	0	0	1	0	0	0.4320	0.7179	0.8012	0.6791	0.5061	0.2160	0.5620	
0	1	1	0	0	0	0.0600	0.2005	0.6630	0.6909	0.4131	0.6858	0.4558	(c)
1	0	1	0	0	0	0.3105	0.4972	0.2104	0.5307	0.4184	0.7832	0.4587	
1	1	0	0	0	0	0.2357	0.4905	0.2667	0.5463	0.4970	0.0719	0.5063	
0	0	0	1	1	1	0.2382	0.2866	0.0311	0.5398	0.6185	0.3926	0.3555	
0	0	1	0	1	1	0.9850	0.8725	0.0650	0.1339	0.8286	0.8501	0.6114	
0	0	1	1	0	1	0.0381	0.0020	0.3848	0.6385	0.1798	0.5928	0.3135	
0	0	1	1	1	0	0.0349	0.0406	0.8821	0.6820	0.1527	0.1949	0.3400	
0	1	0	0	1	1	0.9818	0.2327	0.1004	0.8767	0.8257	0.1579	0.5456	
0	1	0	1	0	1	0.2619	0.9482	0.7321	0.1395	0.1442	0.4807	0.4398	
0	1	0	1	1	0	0.3333	0.5253	0.9647	0.7566	0.1623	0.9693	0.6204	
1	0	0	0	1	1	0.1059	0.0034	0.9498	0.8721	0.1200	0.0776	0.3694	
1	0	0	1	0	1	0.2644	0.1873	0.1889	0.3487	0.1209	0.5796	0.2835	
1	0	0	1	1	0	0.1681	0.8944	0.3040	0.9451	0.8868	0.1167	0.5602	
0	1	1	0	0	1	0.0355	0.1707	0.3687	0.7003	0.8783	0.0096	0.3680	
0	1	1	0	1	0	0.7912	0.1336	0.8704	0.0550	0.6818	0.5826	0.5096	
0	1	1	1	0	0	0.3017	0.1339	0.5458	0.0940	0.4113	0.3252	0.2966	
1	0	1	0	0	1	0.5642	0.5740	0.9024	0.5879	0.9160	0.4144	0.6579	
1	0	1	0	1	0	0.3405	0.1884	0.0092	0.9740	0.5396	0.6047	0.4576	
**a_1_**	**a_2_**	**a_3_**	**a_4_**	**a_5_**	**a_6_**	**f_1_**	**f_2_**	**f_3_**	**f_4_**	**f_5_**	**f_6_**	**F**	
1	0	1	1	0	0	0.1034	0.0965	0.5319	0.2718	0.3936	0.3031	0.2818	
1	1	0	0	0	1	0.0574	0.6499	0.8059	0.0250	0.9335	0.9274	0.5449	
1	1	0	0	1	0	0.2233	0.4043	0.6943	0.8878	0.3928	0.5909	0.5403	
1	1	0	1	0	0	0.6356	0.3956	0.3065	0.6027	0.2874	0.7046	0.4940	
1	1	1	0	0	0	0.8611	0.5306	0.7612	0.2031	0.4915	0.0385	0.4782	(d)
0	0	1	1	1	1	0.3131	0.0464	0.4932	0.2103	0.1307	0.0279	0.3060	
0	1	0	1	1	1	0.1426	0.6688	0.7408	0.1727	0.1627	0.8019	0.4368	
1	0	0	1	1	1	0.0456	0.9952	0.5782	0.7781	0.9560	0.2162	0.4946	
0	1	1	0	1	1	0.3505	0.9309	0.4500	0.1651	0.3388	0.1961	0.3975	
1	0	1	0	1	1	0.0983	0.7039	0.4154	0.9007	0.6736	0.1105	0.4924	
1	1	0	0	1	1	0.7581	0.0250	0.6700	0.8932	0.4175	0.1692	0.5057	
0	1	1	1	0	1	0.0635	0.4125	0.7375	0.3116	0.9059	0.7501	0.5190	
1	0	1	1	0	1	0.3159	0.0187	0.9994	0.3774	0.5740	0.1758	0.4100	
1	1	0	1	0	1	0.3301	0.8663	0.7155	0.3774	0.6545	0.7774	0.6099	
0	1	1	1	1	0	0.7393	0.1859	0.5557	0.8887	0.5421	0.2327	0.5388	
1	0	1	1	1	0	0.8041	0.6003	0.6681	0.4699	0.3208	0.1615	0.5075	
1	1	0	1	1	0	0.9223	0.6873	0.2565	0.6737	0.7160	0.7828	0.5760	
1	1	1	0	0	1	0.7628	0.2460	0.8319	0.9456	0.6548	0.7953	0.7153	(e)
1	1	1	0	1	0	0.0907	0.3601	0.2190	0.7766	0.1938	0.5627	0.3764	
1	1	1	1	0	0	0.5653	0.4253	0.6097	0.0901	0.6707	0.8776	0.5270	
0	1	1	1	1	1	0.0599	0.7584	0.2032	0.4657	0.2394	0.9716	0.4450	
1	0	1	1	1	1	0.8842	0.8518	0.0726	0.7531	0.6258	0.7244	0.6073	
1	1	0	1	1	1	0.1897	0.5880	0.7039	0.0549	0.7979	0.1568	0.4026	
1	1	1	0	1	1	0.8689	0.7059	0.4294	0.7165	0.7862	0.8735	0.7315	(f)
1	1	1	1	0	1	0.7610	0.0442	0.4426	0.3836	0.2056	0.3096	0.3641	
1	1	1	1	1	0	0.1868	0.9939	0.7246	0.3898	0.6835	0.1443	0.5134	
1	1	1	1	1	1	0.8953	0.9262	0.4466	0.9353	0.4430	0.9112	0.7665	(g)

## Data Availability

In the results section, data supporting reported results can be found.
